# Adiposity Predicts Cognitive Decline in Older Persons with Diabetes: A 2-Year Follow-Up

**DOI:** 10.1371/journal.pone.0010333

**Published:** 2010-04-23

**Authors:** Angela Marie Abbatecola, Fabrizia Lattanzio, Liana Spazzafumo, Anna Maria Molinari, Michele Cioffi, Raffaele Canonico, Luigi DiCioccio, Giuseppe Paolisso

**Affiliations:** 1 Scientific Direction, Italian National Research Center on Aging (INRCA), Ancona, Italy; 2 Statistic and Biometry Center, Department of Gerontological Research, Italian National Institute on Aging, Ancona, Italy; 3 Department of General Pathology, Second University of Naples, Naples, Italy; 4 Department of Geriatric Medicine and Metabolic Diseases, Second University of Naples, Naples, Italy; 5 Department of Geriatric Medicine, Santa Scolastica Hospital, Cassino, Italy; University of Tor Vergata, Italy

## Abstract

**Background:**

The mechanisms related to cognitive impairment in older persons with Type 2 diabetes (DM) remains unclear. We tested if adiposity parameters and body fat distribution could predict cognitive decline in older persons with DM vs. normal glucose tolerance (NGT).

**Methodology:**

693 older persons with no dementia were enrolled: 253 with DM in good metabolic control; 440 with NGT (age range:65–85 years). Longitudinal study comparing DM and NGT individuals according to the association of baseline adiposity parameters (body mass index (BMI), waist-hip-ratio (WHR), waist circumference (WC) and total body fat mass) to cognitive change (Mini Mental State Examination (MMSE), a composite score of executive and attention functioning (CCS) over time.

**Findings:**

At baseline, in DM participants, MMSE correlated with WHR (β = −0.240; p = 0.043), WC (β = −0.264; p = 0.041) while CCS correlated with WHR (β = −0.238; p = 0.041), WC (β = −0.326; p = 0.013) after adjusting for confounders. In NGT subjects, no significant correlations were found among any adiposity parameters and MMSE, while CCS was associated with WHR (β = −0.194; p = 0.036) and WC (β = −0.210; p = 0.024). Participants with DM in the 3^rd^ tertile of total fat mass showed the greatest decline in cognitive performance compared to those in 1^st^ tertile (tests for trend: MMSE(p = 0.007), CCS(p = 0.003)). Logistic regression models showed that 3^rd^ vs. 1^st^ tertile of total fat mass, WHR, and WC predicted an almost two-fold decline in cognitive function in DM subjects at 2^nd^ yr (OR 1.68, 95%IC 1.08–3.52).

**Conclusions:**

Total fat mass and central adiposity predict an increased risk for cognitive decline in older person with DM.

## Introduction

Type 2 diabetes mellitus(DM) has been consistently associated with a higher risk of cognitive decline, especially in older persons[1]. Such individuals are almost two times more likely to experience cognitive decline and dementia compared to those with normal glucose tolerance (NGT)[2]. Such decline maybe in part due to an array of tissue response from chronic hyperglycemia[3], postprandial glucose fluctuations[4], advanced glycosylated end-products[5] and altered insulin action[6]. Even though the underlying mechanisms explaining the relationship between diabetes and cognitive decline are still unclear, the presence of increased visceral fat tissue in older diabetics is no longer considered an inert bystander, but an active endocrine organ capable of activating a pro-inflammatory network. Adipose inflammatory proteins have been indicated in diabetic complications leading to hypertension, cardiovascular disease and metabolic syndrome[7]. Considering that older diabetics are at a significantly higher risk of cognitive disability, a further role of changes in body composition, in particular adipose tissue distribution on cognitive decline may be hypothesized.

There is a growing body of literature regarding body fat distribution and cognitive decline in older persons, however overall findings have been controversial. A recent study showed that central obesity was not associated with cognitive decline in older men, while it was associated with a decline in cognitive function in older women[8], while another study underlined that both Waist circumference(WC) and Waist-hip-ratio(WHR) were independent predictors of cognitive function in both older men and women[9]. There is also recent data demonstrating that weight gain during middle age is associated an increased risk for Alzheimer's disease(AD) in both men and women in old age, while central obesity predicts an increased risk for AD in women only[10]–[11]. Data from the Framingham Heart study showed that in both obesity and hypertension were independent predictors of cognitive decline in men, while hypertensive obese men performed more poorly on cognitive tests than men that were either obese or hypertensive alone[12]. Considering that fat tissue distribution seems to play a more detrimental role on diverse health outcomes during type 2 diabetes, an impact on cognitive decline cannot be ruled out. Indeed, data regarding a predictive role of fat tissue distribution on cognitive decline in older persons with type 2 diabetes is lacking.

To address the hypothesis that body fat tissue distribution in older persons with diabetes may predict a greater decline in cognitive performance, we examined the relationship between adiposity parameters at baseline and the risk of decline on cognitive performance over a two year observation period in a large group of older persons with DM and NGT.

## Results

**Figure 1:**
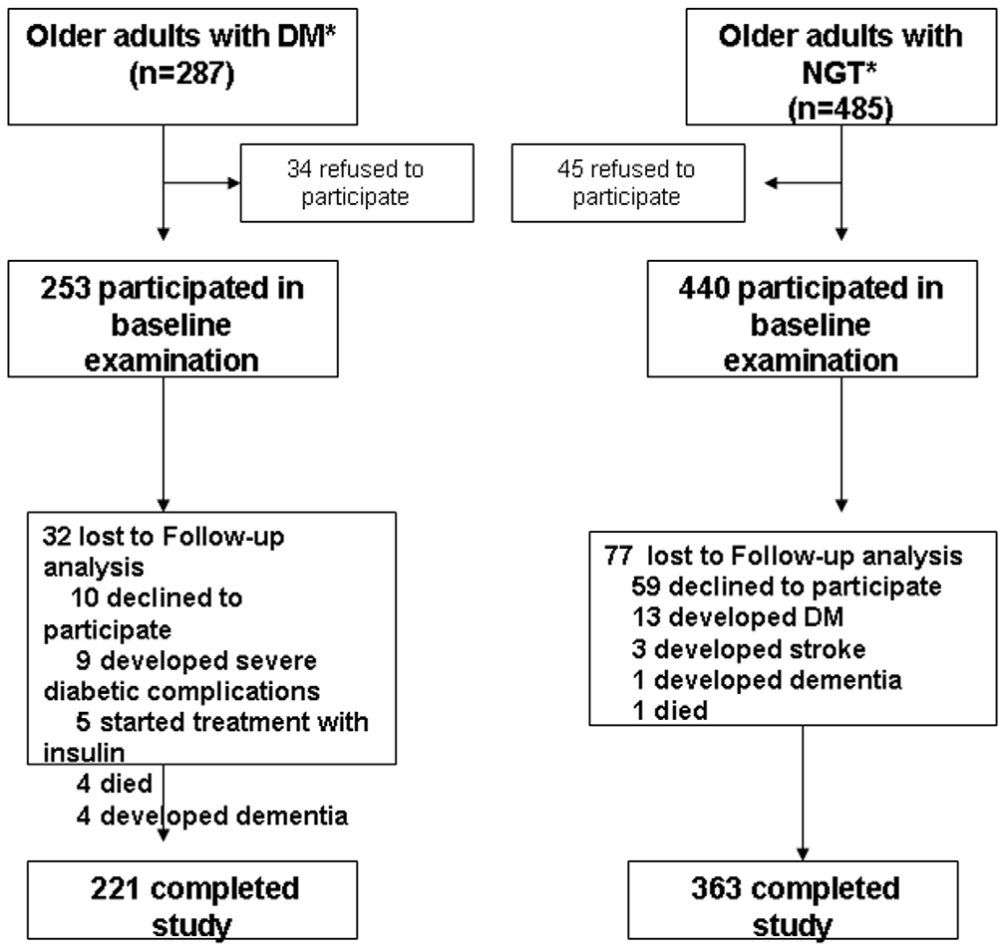
Figure 1 depicts patient recruitment and follow-up diagram.

A total of 221 participants with DM (87%) and 363 with NGT (83%) completed study protocol(Figure 1).

### Baseline data

Study population clinical characteristics are reported in Table 1. The mean (SD) age of the participants at baseline was 73.6(2.8) years. Compared with participants with NGT (n = 440), those with DM(n = 253) were heavier, had greater central fat distribution, more elevated IL-6 and FPG levels as well as a higher percentage of hypertension(Table 1). There were no significant differences among neuropsychological test scores between groups at baseline. In those with DM, we also a significant correlation between total fat mass and HbA1c levels (r = 0.2100; p = 0.009).

**Table 1:** Population characteristics at baseline (n = 693).

Characteristic	NGT (n = 440)	Type 2 Diabetes (n = 253)	p value
Age, yrs	74±6	76±7	0.203
Gender (M/F)			
Education, yrs	5.7±3.8	6.3±3.3	0.649
Hypertension (%)	310 (71%)	201 (79%)	0.129
IMT, mm	0.65±0.2	0.73±0.1*	0.004
SBP (mmHg)	145±19	155±21	0.020
DBP (mmHg)	88±9	92±11	0.105
BMI (kg/m^2^)	26.3±3.7	27.2±3.3*	0.033
WC (cm)	89.7±11.1	91.1±9.6*	0.046
WHR	0.89±0.07	0.91±0.08*	0.050
Total cholesterol (mmol/l)	4.1±0.2	5.2±0.4	0.004
HDL cholesterol (mmol/l)	1.42±0.39	1.28±0.10	<0.001
LDL cholesterol (mmol/l)	2.80±0.24	3.11±0.31	<0.001
Triglycerides (mmol/l)	1.37±0.83	1.95±0.38	<0.001
Total fat mass (kg)	25.7±6.8	26.0±8.6	0.110
FPG (mmol/l)	4.8±0.4	9.0±0.4	<0.001
HbA1c (%)		6.6±0.3	
IL-6 (pmol/l)	2.2±2.1	2.6±2.9	0.006
MMSE	26.1±2.5	25.3±2.2	0.823
TMT-A, sec	63±46	60±39	0.794
TMT-B, sec	163±71	158±93	0.718
DIFFBA, sec	98±68	102±53	0.626
VF	26.4±4.2	25.9±4.8	0.688
DSP-forward	6.8±1.1	6.6±1.0	0.721
DSP-backward	5.1±0.7	4.9±0.8	0.783
Depression score	12.8±2.3	13.9±3.6	0.075
Duration of diabetes, y		9.8±5.9	
Antidiabetic oral agents (%)		78%	
Diet only (%)		22%	

Note: BMI = body mass index; WC = Waist circumference; WHR = waist-hip-ratio; MMSE = Mini-Mental State Examination; SBP = systolic blood pressure, DBP = diastolic blood pressure.

TMT = Trail Making Test; DIFF = Difference; DSP = Digit Span; VF = Verbal fluency.

*p<0.05.

Multivariate linear models testing independent associations among adiposity parameters (BMI, WHR, WC, Total fat mass), chronic inflammation(IL-6) and cognitive tests (MMSE, CCS) showed that MMSE was associated with WHR (β = −0.240;p = 0.043), WC (β = −0.264;p = 0.041) and IL-6 concentrations(β = −0.172;p = 0.014), while CCS was associated with WHR(β = −0.238;p = 0.041), WC(β = −0.326;p = 0.013), IL-6 concentrations(β = −0.188;p = 0.007), and total fat mass(β = −0.272;p = 0.033) after adjusting for age, sex and years of education in DM participants. In NGT participants, no significant correlations were found among any adiposity parameters and cognitive test scores on MMSE(data not shown), while CCS was independently correlated with WHR(β = −0.194;p = 0.036) and WC(β = −0.210;p = 0.024) at baseline.

**Figure 2:**
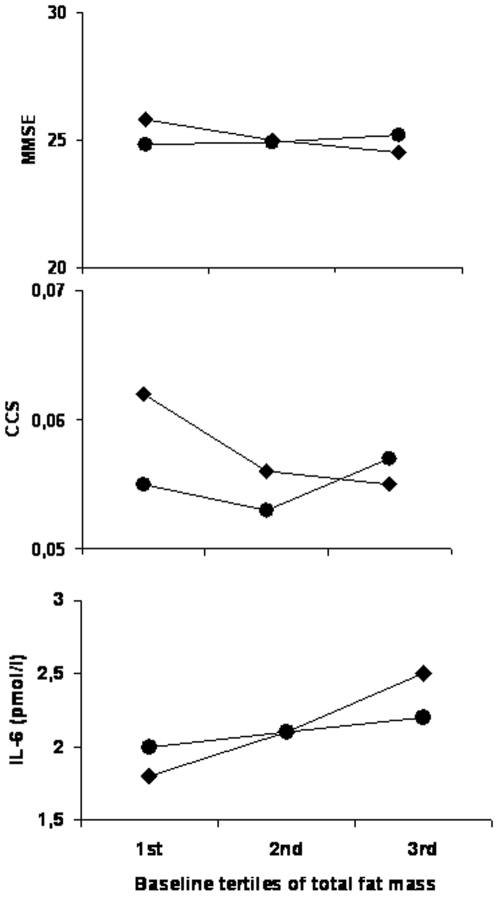
Figure 2 shows MMSE scores, CCS and IL-6 levels across baseline tertiles of total fat mass in persons with DM and NGT; (p for trend <0.05 in DM only for all figures). (black diamonds: DM; black circles: NGT).

Participants with DM in the upper tertile of baseline total fat mass had significantly lower MMSE and CCS scores(Figure 2a–2b) and significantly higher plasma levels of IL-6 compared to those in lower tertiles(Figure 2). Such distribution across total fat mass tertiles was not found in those participants with NGT(Figure 2).

### Follow-up data

At the 2^nd^ year of follow up, DM subjects had gluco-metabolic parameters comparable to baseline ones (HbA1c (6.6±0.3 vs. 6.8±0.2,p = NS) and FPG (8.1±0.4 vs. 8.4±0.3 mmol/l), despite an increase in BMI (27.1±3.3 vs. 28.4±3.0;p = 0.002),WHR (0.91±0.08 vs. 0.93±0.09), Total fat mass (26.0±8.6 vs. 26.4±8.9 kg; p = 0.028) and WC (91.1±9.6 vs. 93.2±0.7 cm; p = 0.002). A worsening degree of cognitive performance on MMSE (25.2±1.1 vs. 24.6±2.2;p<0.04) and CCS (0.055±0.07 vs. 0.041±0.03;p<0.02) was found. In NGT participants, we also found a significant increase in total body fat (25.7±6.8 vs. 27.2±7.2 kg; p = 0.023), BMI (26.3±3.7 vs. 27.2±3.4 kg/m^2^; p = 0.012), WC (89.7±11.1 vs. 91.2±9.8 cm; p<0.001), but without any significant change in cognitive performance compared to baseline values (MMSE:26.2±1.1 vs. 25.9±1.2;p = NS; CCS (0.060±0.06 vs. 0.052±0.03;p = NS).

Over 2 years of follow-up, in DM participants in the 3^rd^ baseline tertile of total fat mass had a greater decline in MMSE(p for trend = 0.007) and CCS(p for trend = 0.003) than those in the 1^st^ tertile. Such trend was also observed in those with NGT, but did not reach statistical significance on either the MMSE or the CCS.

Logistic regression models were used to test the hypothesis that the adiposity parameters would be associated with a higher risk of developing poor cognitive decline(MMSE ≤24) at the 2^nd^ year of follow-up in older persons with DM(Table 2). We found that baseline WC, WHR and the 3^rd^ vs. 1^st^ tertile of total fat mass were significant risk factors for poor cognitive scoring(Table 2).

**Table 2:** Odds ratio and 95% confidence intervals for cognitive impairment (MMSE <24) at follow-up by adiposity measures in older persons with type 2 diabetes (n = 221).

		Unadjusted			Model 1			Model 2	
	OR	95% CI	p	OR	95% CI	p	OR	95% CI	P
**BMI (kg/m^2^)**	0.13	0.76–1.01	0.13	0.29	0.75–1.10	0.29	0.36	0.77–1.10	0.360
**WHR** *	1.42	1.34–3.11	0.04	1.11	1.01–1.12	0.04	1.13	1.15–2.08	0.050
**WC** * **(cm)**	1.81	1.21–3.51	0.03	1.33	1.21–2.89	0.04	1.23	1.09–1.41	0.008
**Total fat mass (kg)**									
**Tertile 1**	1.00	-	-	1.00	-	-	1.00	-	-
**Tertile 2**	1.41	0.70–2.78	0.47	1.26	0.65–2.41	0.46	1.17	0.54–2.39	0.64
**Tertile 3**	2.20	1.23–4.32	0.02	1.92	1.2–3.71	0.04	1.68	1.08–3.52	0.02

**Model 1** included the following confounders: age, gender, yrs of education, physical activity, depression.

**Model 2 = **Model 1 + BMI, hypertension, smoking IMT, Hb1Ac, CV-PPG, IL-6, Drug vs. diet ^§^

§ see methods.

*WHR and WC were entered separately in models.

In a similar analysis testing the independent relationship between CCS, as dependent and continuous variable, baseline adiposity measures such as, WHR, WC and total fat mass values were independent determinants after adjusting for baseline CCS and multiple confounders in DM patients(Table 3). Considering that baseline CCS was also independently associated with WHR and WC, we performed the same analyses in those with NGT. In this latter group, no significant correlations among CCS with any baseline adiposity parameter at the 2^nd^ year of follow-up were observed(data not shown).

**Table 3:** Multivariate linear models testing the independent relationship between CCS, as dependent variable, and adiposity measures according to diabetes status.

	Unadjusted		Model 1		Model 2	
	β (SE)	p	β (SE)	p	β (SE)	p
**DM**						
**BMI (kg/m^2^)**	−0.021 (0.023)	0.349	−0.021 (0.022)	0.386	−0.020 (0.021)	0.394
**WHR** *	−2.741 (1.001)	0.014	−2.264 (0.955)	0.042	−2.358 (1.065)	0.050
**WC** * **(cm)**	−0.018 (0.007)	0.009	−0.020 (0.010)	0.020	−0.019 (0.010)	0.029
**Total fat mass**						
**Tertile 1**	Ref	-	Ref	-	Ref	-
**Tertile 2**	−1.771 (1.410)	0.222	−0.858 (0.571)	0.370	−0.668 (0.546)	0.532
**Tertile 3**	−1.237 (0.329)	0.001	−1.085 (0.295)	0.002	−0.887 (0.279)	0.004

**Model 1** included the following confounders: age, gender, yrs of education, physical activity, depression.

**Model 2 = **Model 1 + BMI, hypertension, smoking IMT, Hb1Ac, CV-PPG, IL-6, Drug vs. diet ^§^.

§ see methods.

*WHR and WC were entered separately in models.

## Discussion

The present study provides evidence that adiposity measures of total fat mass and central fat distribution are associated with a decline in cognitive performance in overweight older persons with DM but not in those with NGT. In particular, we found that older persons with DM in the 3^rd^ tertile of total fat mass at baseline were associated with an approximately two-fold risk of a global cognitive performance decline after 2 years of follow-up. Furthermore, in those with greater central adiposity, an almost 1.5 risk was found for global cognitive decline was found. The CCS score which represents attention and executive functioning, putative of cerebral frontal lobe functioning was also associated with the same adiposity parameters, including the 3^rd^ vs. 1^st^ tertile of total fat mass, WC and WHR over time. These above findings were found independently of age, sex, chronic inflammation, hypertension, metabolic control and years of formal education.

Data in the literature regarding body fat distribution and cognition in older persons remains controversial. While, one study showed that increased central obesity was associated with cognitive performance decline in women[8] others showed central obesity to be associated with cognitive decline in both men and women[9]. There is also strong evidence that the relationship between central adiposity and hypertension may explain lower results on neuropsychological test scores[12], [13]. This synergistic effect of central adiposity and hypertension may be linked to a greater pro-inflammatory state, insulin resistance and cardiovascular risk factors. Indeed our study design excluded older individuals with severe hypertension and/or coronary heart disease and our data reveal that both total fat mass and central adiposity were significantly associated with cognitive decline independently of the presence of hypertension and intimal media thickness.

The risk of dementia in older persons with DM is significantly higher than in those with NGT[2] and numerous investigations have been undertaken to determine the underlying mechanisms for such risk. In particular, recent studies have found an impact of metabolic control during diabetes including HbA1C, FPG, PPG on cognitive performance decline in older persons[3]–[4], [14]. Nevertheless whether a possible role of fat tissue per se and/or body composition may directly impact cognitive performance in older diabetics is lacking. Most studies have linked obesity to a higher degree of insulin resistance which is known to have an important role on cognitive decline. To the best of our knowledge this study is the first to highlight that individuals with DM with higher levels of total fat mass and central adiposity distribution are at higher risk for cognitive decline over time, while such parameters do not seem to have an impact on cognitive performance in NGT subjects. These findings indicate that markers of central fat distribution which are widely known to reflect visceral fat content can not be underestimated when evaluating cognitive functioning in older persons with DM, especially for global, executive and attention functioning. Furthermore, weight gain in middle age is associated with a higher risk for dementia[15]–[16]. However, in elderly less than 75 years of age, the association between Alzheimer's disease and BMI depicted a U-shape curve, while the risk for dementia decreased in those with higher BMI in older persons over the age of 76 years. Interestingly this same study showed that WC was related to a higher risk of dementia in all ages[17]. Furthermore, weight change does not seem to vary significantly in older persons, while tissue fat content is constantly increasing[18]. Therefore, it is not surprising that we did not find any association with BMI status and cognitive functioning considering that aging is characterized by lean body mass loss and adipose tissue increase without weight gain. An important advantage of our study was that we used the BIA method to directly assess total body fat mass and WC for visceral fat, thus overcoming the burden of using only BMI as an adiposity measure in elderly persons.

A possible explanation of why fat tissue seems to be particularly detrimental during diabetes is probably linked to the increased production of pro-inflammatory cytokines[19]. Adipose tissue is an active endocrine organ which produces adipokines know to have both pro and anti-inflammatory properties including adiponectin, leptin, resistin, as well as pro-inflammatory cytokines like tumor necrosis factor-α and Il-6[20]. Leptin levels have been associated with cognitive performance in older individuals[21] In regards to pro-inflammatory cytokines, we found that older persons with DM had significantly higher levels of IL-6 at baseline than those with NGT and even after controlling for IL-6 levels, total fat mass, WC and WHR continued to be risk factors for cognitive decline on MMSE and CCS. We acknowledge that one of the most important anti-inflammatory adipokines, adiponectin, was not measured in these participants and thus an anti-inflammatory role cannot be ruled out. Even though there is recent data highlighting an anti-inflammatory mechanism of adiponectin on cerebral vascular tissue[22], a possible protective action of adiponectin in those with NGT cannot be underestimated. However, the pro-inflammatory status during DM and aging seems to have a more damaging effect on diverse disease states including cognitive decline. Even after adjusting for chronic inflammation, central obesity and total fat mass continued to predict cognitive performance decline.

Our findings demonstrate that total fat mass and central adiposity distribution are independent risk factors for cognitive decline in older persons with DM, thus implicating that a direct role may be played by visceral fat tissue on cognitive performance. Future intervention trials testing if an improvement of such parameters are necessary to determine their impact on cognition in older diabetics.

## Materials and Methods

### Ethics Statement

All subjects gave their informed consent before participating in the study, which the ethical committee of our institution approved

### Study Population

Two hundred and fifty three older persons (age range from 65–85 yrs) with DM in anti-diabetic oral or diet controlled treatment and four hundred and forty with NGT volunteered for the study. Data collection started in September 2002 and was completed in January 2009(Figure 1).All participants were selected from university out-patient offices(n = 2) after the exclusion of severe macro- and microangiopathy, coronary heart disease, heart failure, renal failure, depression, dementia and/or any signs of edema or dehydration. No subject was using drugs that could affect water-mineral homeostasis. An oral glucose tolerance test(75 g glucose) was used to test for DM in those individuals with fasting glucose levels between 100 mg/dl and 124 mg/dl.

Each participant underwent an in-person interview of general health and function, including medical history three weeks prior to study entry with training for the correct use of self blood glucose monitoring instrument in those with DM. Two weeks prior to the baseline and follow-up visits, DM participants were asked to record his/her self-monitoring of blood glucose levels at fasting and 2 hours after lunch and dinner two times a week. During visits, DM participants were administered a standard main meal in accordance with their individual dietary pattern and 2-hour post-prandial blood samples were drawn and stored for determinations of plasma metabolites. In all participants, a standard clinical examination, biochemical parameters, adiposity measurements, physical and neuropsychological assessments were assessed at baseline and the 2^nd^yr follow-up visit.

### Anthropometric and Adiposity Determinations

Weight, height, WC and WHR were measured using standard techniques. Body mass index(BMI) was calculated as weight(kg) divided by height(m) squared.

Total fat mass and total fat percentage were calculated using the Body Composition Analyzer Scale BF-350 (Tanita Corporation) which uses leg-to-leg bioelectrical impedance analysis(BIA). This technique provides a valid measure of total body fat in older adults compared to the traditional hand-to-foot BIA method [23]–[24].

### Neuropsychological battery

All participants underwent the following tests of cognitive performance: Mini Mental State Examination(MMSE), the Verbal Fluency(VF), Digit span (DSp) forward and backward, Trail Making Test A(TMT-A) and Trail Making Test B(TMT-B). All cognitive evaluations were made by trained physicians who were unaware of study protocol.

The MMSE was used to assess for global cognitive function[25]. The TMT is visuomotor speeded task that consists of two parts: TMT-A and TMT-B. TMT-A, visual scanning test, requires one to draw a line connecting consecutive numbers from 1 to 25. TMT-B, adds cognitive flexibility to TMT-A and requires one to draw a line connecting numbers and letters in alternating sequence. The difference between the two scores, TMT-B minus TMT-A, provides a measure of cognitive efficiency[26]. Verbal fluency test require participants to generate as many words as possible in 1 minute for a given letter (F,A,S)[25].

The Wechsler Adult Intelligence Scale-Revised Digit Span is a measure of mental tracking as well as, of brief storage and mental manipulation[25].

Depression was evaluated using the Center for Epidemiological Studies Depression Scale(CES-D) [27].

### Metabolic determinations

Serum glucose, lipid, and lipoprotein were quantified from fresh samples drawn after a least 12 hours fasting. Serum glucose level was determined by an enzymatic colorimetric assay using a modified glucose oxidase-peroxidase method and a Roche-Hitachi 917 analyzer. Commercial enzymatic tests were used for determining serum total and high-density lipoprotein cholesterol and triglyceride (Roche Diagnostics, Germany) levels. Serum low-density lipoprotein cholesterol levels were calculated by the Friedewald formula [28].

Serum samples for Interleukin 6(IL-6) levels were stored at −80°C until assay. Serum concentrations of IL-6 were determined in duplicate manner with commercially available kits(R&D Systems). Intra-assay and interassay coefficients of variation were 3.9% and 5.9%.

### Physical activity

A modified version of the European Prospective Investigation on Cancer and Nutrition physical activity questionnaire was performed[29]. The level of physical activity ranged from 1(bedridden) to 7(high intense physical activity).

### Other covariates

Smoking habit was categorized according to cigarette use as current smoker and never smoked. Blood pressure was recorded by mercury sphygmomanometer on three occasions separated by 2 minute intervals and the average of the last two measures was used for analysis. Hypertension was defined according to the following criteria as: systolic arterial pressure >140 mmHg and diastolic arterial pressure >90 mmHg and/or taking antihypertensive medication.

Carotid ultrasound evaluation was performed by two trained investigators who were unaware of study protocol.

In those participants with DM, a dichotomous drug variable was created in which 1 was equal to the presence of an oral anti-diabetic oral treatment or 0 equal to diet controlled treatment.

### Statistical Analysis

Statistical analyses were performed using SPSS software(Chicago,IL). All data are represented as mean±standard deviation(SD). To approximate normal distributions, log-transformed values for plasma IL-6 and triglycerides, were used in analyses and back transformed for data presentation. Analysis of variance(ANOVA) test was used to evaluate the differences in clinical and metabolic characteristics between those with DM and NGT and across total body fat tertiles. Multivariate linear models were created to test the independent associations among adiposity parameters and cognitive test scores at baseline after adjusting for age, sex and years of education. Pearson coefficient correlations were performed to test the association between Hb1Ac and total fat mass in those with type 2 diabetes.

As previously reported[6], a cluster analysis was created by using the squared sum of z scores, in order to obtain an overall value of attention and executive function test. The cognition composite score of attention and executive functions(CCS) was calculated as sum of the squared z scores of TMT-A, TMT-B, DIFF B-A, Dsp-Forward, Dsp- Backward, and VF.

Multivariate linear regressions models were performed at baseline separately in DM and NGT participants to test the relationship between cognitive test scores(CCS and MMSE) and tertiles of total fat mass, total fat mass after adjusting multiple confounders. For each patient with DM, the levels of mean fasting plasma glucose(M-FPG), coefficient variation(CV) of fasting plasma glucose(CV-FPG), and CV of post-prandial glucose(CV-PPG) were computed[30]. Glucose levels used in the analysis were those registered by self monitoring blood glucose, as well as those obtained during the follow-up visit. 24-hour CV were calculated using two determinations for PPG (2 hour post –lunch and –dinner glucose levels taken two times a week and the PPG following the standard meal during the examination. ANOVA tested the association between the change in cognitive performance as dependent variable across tertiles of baseline total fat mass. Logistic regression models were created and performed separately according to diabetes to assess the risk of developing poor cognitive performance(MMSE ≤24) at follow-up according to adiposity measures after adjusting for confounders. A multivariate linear analysis was used with the CCS as dependent variable to test the independent relationship with diverse adiposity measures after adjusting for confounders.
